# Safety Assessment of *Aspergillus cristatus* CCNH008 for Potential Use in Food and Health Applications

**DOI:** 10.3390/foods15122066

**Published:** 2026-06-08

**Authors:** Zishan Jiao, Jiahao Huang, Juan Yang, Xinyi Chen, Xiaowei Zheng

**Affiliations:** Nutrition & Health Research Institute, COFCO Corporation, Beijing 102209, China; jiaozishan@cofco.com (Z.J.); huangjiahao@cofco.com (J.H.)

**Keywords:** *Aspergillus cristatus*, CCNH008, safety assessment, oral toxicity, genotoxicity

## Abstract

*Aspergillus cristatus* is a key microorganism involved in the fermentation of Fu brick tea, but systematic strain-level safety data remain limited despite its long history of use in dark tea production. In this study, the toxicological safety of *A. cristatus* CCNH008 was assessed through acute oral toxicity assay, a 90-day repeated oral toxicity study with a recovery period and genotoxicity assays. In acute oral toxicity tests, CCNH008 caused no mortality, no adverse clinical signs, and no treatment-related effects on bodyweight or pathology in mice or rats, with an LD_50_ greater than 10 g/kg bodyweight. In the 90-day repeated oral toxicity study in rats, the oral administration of CCNH008 at doses up to 1.67 g/kg bodyweight/day produced no treatment-related changes in clinical signs, bodyweight, food consumption, hematological or biochemical parameters, urinalysis, organ weights, or histopathology, including during a 28-day recovery period. Genotoxicity was evaluated using a bacterial reverse mutation assay, an in vitro mammalian chromosome aberration test, and an in vivo mammalian erythrocyte micronucleus test; no mutagenic or clastogenic effects were observed. Collectively, these findings demonstrate that CCNH008 shows no evidence of acute, subchronic oral toxicity, or genotoxicity, providing important strain-level safety evidence to support its potential application in food-related products.

## 1. Introduction

Fu brick tea (Fuzhuan tea), a traditional post-fermented dark tea originating in China, is characterized by the growth of “golden flowers” on the tea surface, a feature widely regarded as an important indicator of product quality [1,2,3]. As a representative microbial-fermented tea, Fu brick tea has attracted increasing attention not only for its unique sensory properties but also for its potential health-related benefits. China is the world’s largest producer of dark tea, with an annual output of approximately 473,200 tons in 2024 [4]. Among dark teas, Fu brick tea is mainly produced in Jingyang (Shaanxi Province) and Yiyang (Hunan Province), with an annual output of about 30,000 tons [5,6]. In parallel, the fermented food sector has continued to expand rapidly worldwide. At the same time, the fermented food market continues to grow worldwide, reflecting increasing consumer interest in products with functional and wellness-related benefits [7]. Against this backdrop, the safety of microorganisms used in fermentation has become a central issue for both food innovation and industrial development.

The genus *Aspergillus* occupies a prominent position in food biotechnology and industrial fermentation. Several species, including *A. oryzae* and *A. niger*, are widely used for enzyme production, organic acid manufacture, and food fermentation, and some have been recognized for their long-standing safety history in regulated food applications. For example, *A. niger* is a major industrial strain for citric acid and enzyme production, while *A. terreus* is known for lovastatin production [8,9]. In sum, *Aspergilli* enable the efficient production of flavorings, food additives, and functional foods. However, the genus also includes toxigenic species that may produce aflatoxins, ochratoxin A, and sterigmatocystin [10,11]. Food surveillance studies have shown that *Aspergillus* species are frequently detected in fermented, dried, and stored foods. For example, *A. niger*, *A. flavus*, and other potential toxigenic species have been reported as common contaminants in black tea and related products, and aflatoxins have been detected above permissible levels in a substantial proportion of tea samples in some surveys [12,13,14]. These findings highlight that the presence of *Aspergillus* in food matrices does not automatically imply safety and that strain-level assessment is necessary before broader application.

*Aspergillus cristatus* is the key functional microorganism responsible for the formation of the characteristic “golden flower” (Fahua) during the fermentation of Fu brick tea. During this process, the fungus proliferates rapidly and forms abundant golden-yellow cleistothecia, hence its common name, “golden flower fungus” [2,15]. *A. cristatus* and its sexual morph Eurotium cristatum are recognized as the predominant fungal species in dark teas [16]. Previous studies have shown that *A. cristatus* contributes to the formation of tea aroma and taste, including the reduction in bitterness and astringency [17]. In addition, it can adapt to moist and mildly acidic fermentation conditions; under osmotic stress, it can adjust pathways such as tryptophan metabolism and flavonoid biosynthesis to improve antioxidant capacity [18]. Bioactive metabolites from *A. cristatus*, including anthraquinones, polysaccharides, and volatile compounds, have been reported to exhibit anti-inflammatory, immunomodulatory, gut-protective, and skin-related activities [19,20,21,22]. These findings suggest its potential application in functional food development.

Although *A. cristatus* is generally considered to be a benign fungi, commonly associated with traditional fermented foods [1,2,23], systematic toxicological data at the strain level remain limited. In particular, information on acute toxicity, repeated-dose toxicity, and genotoxicity of *A. cristatus* used in Fu brick tea fermentation is still insufficient. The *A. cristatus* strain used in this study, designated CCNH008, was isolated from the golden flower fermentation process of dark tea produced by a century-old tea factory in Hunan Province, China. Identification was based on internal transcribed spacer (ITS) sequencing confirmed the strain as *A. cristatus*. The strain has been deposited at the China General Microbiological Culture Collection Center (CGMCC) under the accession number CGMCC No. 41727. In this study, acute oral toxicity tests, a 90-day oral toxicity study, bacterial reverse mutation assays, mammalian erythrocyte micronucleus tests, and in vitro mammalian cell chromosomal aberration assays were conducted.

## 2. Methods

Experimental reagent and Animals: Cyclophosphamide (CP) was purchased from Sigma-Aldrich (St. Louis, MO, USA) (batch No. WXBD0289V). The S9 metabolic activation system was obtained from Molecular Toxicology Inc. (Boone, NC, USA), USA (batch No. 4807). Mitomycin C (MMC) (batch No. GC12353) was purchased from GLPBIO. (Montclair, CA, USA) ICR-191 (batch No. SLCR1359), 4-nitroquinoline-N-oxide (batch No. WXBD8520V), methyl methanesulfonate (batch No. WXBD8272V), 2-aminofluorene (batch No. STBF2358V), 1,8-dihydroxyanthraquinone (batch No. WXBC4791V), and 2-aminoanthracene (batch No. STBJ3963) were all purchased from Sigma-Aldrich. Sodium azide (batch No. 20210122) was obtained from Sinopharm Chemical Reagent Co., Ltd. (Shanghai, China). An automated modular hematology analyzer (XN-1000V [B1], Sysmex) was used for hematological analysis. Hematology reagents were supplied by Sysmex Medical Electronics (Shanghai) Co., Ltd. (Shanghai, China); coagulation assay reagents were provided by Wuhan Seares Biotechnology Co., Ltd. (Wuhan, China); urinalysis reagents were obtained from Roche Diagnostics (Shanghai) Co., Ltd. (Shanghai, China).

The test strain, *A. cristatus* CCNH008, was obtained from the proprietary strain repository of COFCO Nutrition & Health Research Institute Co., Ltd. (Beijing, China). The *Salmonella typhimurium* tester strains TA97a, TA98, TA100, TA102, and TA1535, used in the bacterial reverse mutation assay, were purchased from Molecular Toxicology, Inc. (United States). The Chinese hamster lung (CHL) cell line, employed for the in vitro chromosomal aberration assay, was obtained from Wuhan Punoise Biotechnology Co., Ltd. (Wuhan, China).

Specific-pathogen-free (SPF) Kunming mice were purchased from Shanghai Jiesijie Laboratory Animal Co., Ltd. (Shanghai, China; production license No. SCXK [Shanghai] 2023-0004). SPF Sprague Dawley (SD) rats were obtained from Zhejiang VITONLIVIA Technology Co., Ltd. (Shaoxing, China; production license No. SCXK [Zhe] 2024-0001). All experimental animal procedures were conducted in accordance with institutional guidelines and were approved under the ethical approval for laboratory animals permit (No. IACUC-SL-004-2025, No. IACUC-SL-009-2025, IACUC-SL-012-2025).

Revival and preparation of *Aspergillus cristatus* CCNH008 spore powder: A glycerol stock of *Aspergillus cristatus* CCNH008 stored at −80 °C was revived by aseptically streaking the culture onto potato dextrose agar (PDA) plates using a sterile inoculating loop. The plates were incubated at 25 °C for 6–8 days. After incubation, sterile physiological saline was added to the surface of the agar to gently dislodge the spores, and the resulting spore suspension was collected and transferred into sterile 250 mL Erlenmeyer flasks.

The spore suspension was subsequently inoculated into sterile tea leaves with a moisture content of 50% (*w*/*w*) at an inoculation level of 1% (*w*/*w*). After thorough mixing, the inoculated tea leaves were evenly spread in sterile 1 L Erlenmeyer flasks and incubated at 25 °C. After 2 days of incubation, when light-colored mycelia were observed on the surface of the tea leaves and partial agglomeration occurred, the clumps were gently broken up to ensure uniform growth, followed by continued incubation for an additional 6 days.

Upon completion of cultivation, the mycelial biomass was collected and evenly spread onto trays, then dried in a forced-air oven at 50 ± 5 °C for 24 h. The dried biomass was subsequently ground into a fine powder and passed twice through a 40-mesh sieve to obtain *A. cristatus* CCNH008 spore powder. The viable cell count of the resulting spore powder was determined prior to use.

Acute oral toxicity study: The acute oral toxicity study was conducted in accordance with the National Food Safety Standard of China: Acute Oral Toxicity Test (GB 15193.3-2014) [24]. SPF Kunming mice (10 males and 10 females) and SD rats (10 males and 10 females) were used in this study. All animals underwent a 3-day acclimation period prior to dosing, after which they were weighed and randomly assigned to experimental groups based on bodyweight.

The test article was *A. cristatus* CCNH008 spore powder with a viable count of 3.0 × 10^9^ CFU/g. For dose preparation, 10.002 g of spore powder (for the mouse study) and 40.002 g of spore powder (for the rat study) were accurately weighed and suspended in 1% (*w*/*v*) Tween 80 solution; these were then adjusted to final volumes of 60 mL and 240 mL, respectively, to obtain homogeneous dosing suspensions.

The animals were administered the test suspensions by oral gavage following a split-dose regimen. Within a 24 h period, each animal received three administrations at a dosing volume of 20 mL/kg bodyweight, with a 4 h interval between consecutive doses. After completion of dosing, all animals were observed for a 14-day post-exposure period. Clinical signs, toxicological symptoms, and mortality were monitored and recorded daily, and bodyweights were measured weekly throughout the observation period. At the end of the observation period, all surviving animals were humanely euthanized and subjected to necropsy. Any animals that died during the study were promptly necropsied. Gross pathological examinations were performed on all animals, and tissues exhibiting abnormal findings were further collected for histopathological examination and analysis.

Ninety-day oral toxicity study: The 90-day oral toxicity study was conducted in accordance with the National Food Safety Standard (GB 15193.13-2015) [25]. SPF SD rats of both sexes were used, with 20 animals per sex in each group. Animals were assigned to a high-dose group (1.67 g/kg bodyweight), a mid-dose group (0.84 g/kg bodyweight), a low-dose group (0.42 g/kg bodyweight), and a vehicle control group (1% Tween-80). Additional satellite groups were established for the control and high-dose groups, including an interim blood-sampling group (*n* = 10/sex/group) and a 28-day recovery observation group (*n* = 10/sex/group). Rats were administered the test substance once daily by oral gavage, 6 days per week, for a total duration of 90 consecutive days. All administrations were performed at approximately the same time each day. Throughout the study, clinical signs were observed and recorded daily, while bodyweight and food consumption were measured periodically. At the midpoint of the study, animals in the interim blood-sampling groups underwent urinalysis, hematological, and serum biochemical examinations. At the end of the 90-day exposure period, all animals were subjected to the same evaluations, followed by a complete gross necropsy and histopathological examination. Urinalysis parameters included urine appearance, protein, specific gravity, pH, glucose, and occult blood. Hematological assessments comprised total and differential leukocyte counts, erythrocyte count, hemoglobin concentration, hematocrit, platelet count, prothrombin time (PT), and activated partial thromboplastin time (APTT). Serum biochemical analyses included alanine aminotransferase (ALT), aspartate aminotransferase (AST), γ-glutamyl transferase (GGT), alkaline phosphatase (ALP), blood urea nitrogen (BUN), creatinine (Cr), glucose (Glu), total protein (TP), albumin (Alb), total cholesterol (TC), triglycerides (TG), chloride (Cl^−^), potassium (K^+^), and sodium (Na^+^). Following cessation of dosing, animals in the recovery groups were maintained without treatment for an additional 28 days. At the end of the recovery period, the same sets of urinalysis, hematological, serum biochemical, gross pathological, and histopathological examinations were conducted to assess the reversibility or persistence of any treatment-related effects.

Bacterial reverse mutation assay (Ames test): The bacterial reverse mutation assay (Ames test) was conducted in strict accordance with the National Food Safety Standard of China: Bacterial Reverse Mutation Test (GB 15193.4-2014) [26]. The standard *Salmonella typhimurium* tester strains TA97a, TA98, TA100, TA102, and TA1535 were used. After activation, the strains were cultured in nutrient broth at 37 °C with shaking until reaching a bacterial concentration of 1.1–1.6 × 10^9^ CFU/mL. An in vitro metabolic activation system consisting of an S_9_ mix containing 10% (*v*/*v*) S_9_ fraction was employed where applicable.

Preliminary tests were performed prior to the main assays to evaluate the solubility of CCNH008 spore powder and its potential cytotoxicity to the test strain TA98. The spore powder was suspended in sterile distilled water, serially diluted, and assessed via the plate incorporation method. According to the preliminary test results, the maximum dose for the main assays was set at 2500 μg/plate, with all dose levels designed on this basis.

The main assays were performed using the plate incorporation method. Each experiment included untreated controls, solvent controls (sterile distilled water), and positive controls. In brief, the tester strain culture, test substance solution at each dose level (or the corresponding control), and S_9_ mix (for the +S_9_ treatment groups) were added to the top agar in sequence, mixed fully, and poured onto minimal agar plates. For each dose, three replicate plates were set up for both +S_9_ and −S_9_ conditions. All plates were incubated at 37 °C for 48 h, after which the number of revertant colonies was counted.

Two independent main assays were performed to assess the dose–response relationship. In the first assay, a √10 dose interval was applied (2500, 790, 250, 79, and 25 μg/plate), while in the second assay, a five-fold dose interval was used (2500, 500, 100, 20, and 4 μg/plate). The strain-specific positive mutagens used in the assays are listed in Table 1.

Mammalian erythrocyte micronucleus assay: The mammalian erythrocyte micronucleus assay was conducted in accordance with the National Food Safety Standard-Mammalian Erythrocyte Micronucleus Test (GB 15193.5-2014) [27]. Based on the results of the acute oral toxicity study (LD_50_ > 10 g/kg BW), the maximum test concentration of *A. cristatus* CCNH008 was set at 167 mg/mL in 1% Tween-80. Three dose levels were established: 1.67, 0.84, and 0.42 g/kg BW, respectively. A vehicle control group (1% Tween-80) and a positive control group (cyclophosphamide, 4 mg/mL) were included.

SPF mice were randomly assigned by bodyweight into five groups (*n* = 10 per group, equal numbers of males and females). The test substance was administered by oral gavage twice within 30 h (24 h apart) at a dosing volume of 10 mL/kg BW per administration. At 6 h after the final dosing, animals were euthanized and femoral bone marrow cells were collected. Cell suspensions were prepared in calf serum, smeared on glass slides, fixed with methanol, and stained with Giemsa. For each animal, 2000 polychromatic erythrocytes (PCEs) were scored to determine the micronucleated PCE frequency (‰). In addition, 200 erythrocytes (including PCEs and normochromatic erythrocytes) were counted to calculate the proportion of PCEs (%), which was used to assess potential cytotoxic effects of the test substance on bone marrow hematopoiesis. The proportion of PCEs in total erythrocytes was required to be no less than 20% of that in the vehicle control group to ensure assay validity.

Statistical analyses were performed using SPSS Statistics, version 29.0 (IBM Corp., Armonk, NY, USA) by the Shanghai Municipal Center for Disease Control and Prevention (Shanghai Academy of Preventive Medicine). The frequencies of micronucleated PCEs in each treatment group were analyzed based on a Poisson distribution and compared with the vehicle control group.

In vitro mammalian cell chromosomal aberration assay: The in vitro mammalian cell chromosomal aberration assay was performed in accordance with the National Food Safety Standard of China: In Vitro Mammalian Cell Chromosomal Aberration Test (GB 15193.23-2014) [28] using Chinese hamster lung (CHL) cells. Prior to the main assays, a cytotoxicity pretest was conducted to determine the appropriate dose levels for the formal experiments. Cells were exposed for 4 h to *A. cristatus* CCNH008 at concentrations ranging from 156.3 to 5000 μg/mL under both metabolic activation (+S_9_) and non-activation (−S_9_) conditions. The S_9_ mix consisted of 1 mL S_9_ fraction, 0.2 mL of 0.4 M MgCl_2_, 0.2 mL of 1.65 M KCl, 17.9 mg glucose-6-phosphate (G-6-P), and 30.6 mg nicotinamide adenine dinucleotide phosphate (NADP), with serum-free MEM medium added to a final volume of 10 mL. The components of the positive control are listed in Table 2. Cytotoxicity was evaluated by calculating the mitotic index, and the concentration inducing approximately 50% inhibition of cell division was selected.

Based on the results of the cytotoxicity pretest, three dose levels (1250, 625, and 312.5 μg/mL) were selected for the main assay. CHL cells were treated with the test article for 4 h under both +S_9_ and −S_9_ conditions. Colchicine was added 4 h prior to cell harvest at a final concentration of 1 μg/mL. After conventional slide preparation, 100 well-spread metaphase cells were examined per treatment group. The types of chromosomal aberrations were recorded, and the aberration frequency was calculated. Negative controls (serum-free MEM medium) and appropriate positive controls were included in each experiment.

As no positive results were observed in the initial assays, an additional extended exposure assay without metabolic activation was performed. Cells were treated for 24 h at concentrations of 625, 312.5, and 156.3 μg/mL, as determined from the pretest. The experimental procedures and evaluations were conducted as described above. Statistical analyses were performed using SPSS Statistics, version 29.0 (IBM Corp., Armonk, NY, USA) by the Shanghai Municipal Center for Disease Control and Prevention (Shanghai Academy of Preventive Medicine). The incidence of cells with structural chromosomal aberrations (excluding gaps) was compared between treatment groups and the negative control using the χ^2^ test or Fisher’s exact test, where appropriate. A Cochran–Armitage trend test was applied to assess the presence of a dose–response relationship. All statistical tests were two-sided, and *p* < 0.05 was considered statistically significant.

## 3. Results

**1.**  Acute oral toxicity of strain CCNH008

In the acute oral toxicity study in mice, all animals remained active with normal behavior throughout the observation period after test article administration. The mice showed normal posture, glossy fur, and no clinical signs of toxicity, with no mortality occurring during the study. Bodyweight increased steadily throughout the 14-day observation period for both sexes. Paired *t*-test analysis confirmed significant weight gain from Day 0 to Day 14 (*p* < 0.01, paired *t*-test). The observed weight gain (54.9% for females, 90.2% for males) fell within the normal physiological range for this mouse strain, no treatment-related bodyweight loss or growth retardation was observed (Table 3). At the end of the study, all mice were humanely euthanized for gross necropsy. Macroscopic examination showed no abnormalities in any major organs, and for this reason, histopathological examination was not conducted.

In the rat acute oral toxicity study, transient clinical signs were noted within 0.5–4 h post-dosing, including reduced activity in all animals (10/10 female, 10/10 male), mild ptosis in a small subset of rats (2/10 female, 4/10 male), and salivation (1/10 female, 5/10 male). These signs were temporary, and all rats recovered to normal conditions within 24 h. Compared with Day 0, bodyweights were significantly higher at Day 7 and Day 14 in both female (*p* < 0.01, paired *t*-test) and male (*p* < 0.01, paired *t*-test) rats. The relative weight gain from Day 0 to Day 14 reached 33.3% in females and 72.7% in males. No further treatment-related clinical signs or mortality were observed during the 14-day observation period. All rats exhibited normal growth, with no evidence of bodyweight loss or other toxic effects (Table 4). Upon completion of the observation period, all animals were euthanized and subjected to gross pathological examination. No macroscopic pathological changes were detected in any organs (Table 5); therefore, histopathological evaluation was not conducted.

Based on the results obtained from both mouse and rat studies, *A. cristatus* CCNH008 was classified as practically non-toxic following acute oral exposure.

**2.**  Ninety-Day Oral Toxicity study of strain CCNH008

During the 90-day treatment period, no treatment-related clinical signs or overt toxic symptoms were observed in rats from any dose group. Sporadic statistically significant differences in food consumption, bodyweight, or bodyweight gain were noted in isolated time points, including transient decreases in food intake or bodyweight gain in male rats of certain dose groups between weeks 2 and 7, as well as a decrease in bodyweight in high-dose female rats during the second week of the recovery period (Supplementary Materials, Tables S1–S3). However, these changes did not exhibit a clear dose–response relationship, were not persistent over time, and remained within the range of historical control variability. Therefore, they were considered incidental and not biologically meaningful.

In serum biochemical and hematological assessments conducted during the 90-day oral toxicity study and the subsequent 28-day recovery period, no biologically significant changes were observed in rats across all dose groups. Although a limited number of parameters showed statistically significant differences (compared with control *p* < 0.05), these changes were sporadic and isolated. Specifically, a decrease of approximately 9.1% in albumin (Alb) was observed in high-dose female rats at the interim examination (Supplementary Materials, Table S4); at study termination, slight reductions in Alb were noted in high-dose male rats (3.7%) and mid-dose female rats (9.2%), accompanied by decreased triglyceride (TG) levels in mid- and high-dose female rats and increased sodium (Na) concentrations in low- and high-dose female rats (Supplementary Materials, Table S5). At the end of the recovery period, marginal decreases in total protein (TP; 5.4%) and Alb (5.6%) were observed in high-dose male rats (Supplementary Materials, Table S6). In hematological assessments, an increased neutrophil percentage (NEUT%) was observed in high-dose male rats at the interim examination (Supplementary Materials, Table S7). At study termination, a decrease in monocyte percentage (MONO%) was noted in mid-dose females, and a shortened prothrombin time (PT) was observed in high-dose males (Supplementary Materials, Table S8). During the recovery period, high-dose male rats exhibited an increased lymphocyte percentage (LYMPH%) and a corresponding decrease in NEUT% (*p* < 0.01) (Supplementary Materials, Table S9). Overall, these sporadic and isolated fluctuations were not considered to be indicative of treatment-related toxic effects.

In urinalysis, no statistically significant differences (*p* > 0.05) were observed in any urinary parameters, including appearance, pH, specific gravity, protein, occult blood, or glucose, in male or female rats across all dose groups compared with the vehicle control group (Supplementary Materials, Table S10). These results indicate that, under the conditions of this study, the test substance did not produce any observable adverse effects on renal function or the urinary system in rats.

Gross necropsy and histopathological examinations were conducted on all animals at the end of the 90-day dosing period and after the 28-day recovery observation. No treatment-related abnormalities were observed in the gross appearance or organ weights of major organs across all dose groups compared with the control group (*p* > 0.05) (Supplementary Materials, Tables S11 and S12, Table 6 and Table 7). Comprehensive histopathological examinations were performed on a total of 24 organs and tissues from all animals in the high-dose and control groups. Sporadic and minimal lesions were observed in a small number of animals in both groups (Table 8). Overall, no treatment-related or dose-dependent pathological changes attributable to CCNH008 were observed, and all minor findings were considered incidental background lesions.

**3.**  Genotoxicity Study of strain CCNH008

### 3.1. Ames Test

The preliminary tests demonstrated that the test article exhibited limited solubility in water, dimethyl sulfoxide (DMSO), and acetone, forming suspensions in all solvents evaluated. Sterile distilled water was therefore selected as the preferred solvent. At a dose of 5000 μg/plate, substantial precipitation of *A. cristatus* CCNH008 spore powder was observed, which interfered with accurate colony counting; consequently, 2500 μg/plate was selected as the maximum dose for the main assays.

In the plate incorporation pretest conducted with the tester strain TA98 under both metabolic activation (+S_9_) and non-activation (−S_9_) conditions, the background lawn density and morphology at doses ranging from 625 to 2500 μg/plate were comparable to those of the untreated control, indicating that the test article did not exert cytotoxic effects on the bacteria under the experimental conditions.

In the two independent main assays (Table 9 and Table 10), the numbers of revertant colonies observed for all five tester strains at all dose levels, both in the presence and absence of S_9_ metabolic activation, did not exceed twofold of the corresponding solvent controls and showed no dose–response relationship. Under the conditions of this study, *A. cristatus* CCNH008 spore powder did not induce gene mutations in any of the tested bacterial strains at doses up to the maximum tested concentration of 2500 μg/plate, regardless of metabolic activation.

### 3.2. Mammalian Erythrocyte Micronucleus Assay

To evaluate the in vivo genotoxic potential of *A. cristatus* CCNH008 spore powder, a mammalian erythrocyte micronucleus assay was performed. As shown in Table 11, the frequencies of micronucleated cells (‰) in both male and female mice treated with CCNH008 at doses of 1.67, 0.84, and 0.42 g/kg bodyweight did not differ significantly from those of the solvent control group (*p* > 0.05). In addition, the proportion of polychromatic erythrocytes (PCEs) among total erythrocytes in all treated groups was not less than 20% of that observed in the control group, indicating the absence of bone marrow cytotoxicity.

These results demonstrate that, *A. cristatus* CCNH008 did not induce a significant increase in micronucleus formation in mouse bone marrow erythrocytes, and the in vivo micronucleus test was therefore considered negative.

#### In Vitro Mammalian Cell Chromosomal Aberration Assay

To further assess whether *A. cristatus* CCNH008 could induce chromosomal-level damage, an in vitro mammalian cell chromosomal aberration assay was conducted. The cytotoxicity pretest (Table 12) showed that, following 4 h exposure to CCNH008 spore powder, the mitotic index of CHL cells decreased to approximately 50% at a concentration of 1250 μg/mL under both metabolic activation (+S_9_) and non-activation (−S_9_) conditions. This concentration was therefore selected as the highest dose for the main assays.

In the main assays (Table 13 and Table 14), CHL cells were treated for 4 h in the presence or absence of S_9_ metabolic activation. The results indicated that the frequencies of chromosomal aberrations at all tested concentrations (1250, 625, and 312.5 μg/mL) were not significantly increased compared with the negative control group (*p* > 0.05), regardless of metabolic activation.

Given the negative outcomes observed in the short-term exposure assays, an additional extended exposure assay without metabolic activation was performed. The preliminary test (Table 15) identified 625 μg/mL as the concentration causing approximately 50% inhibition of the mitotic index following 24 h treatment. Under these conditions (Table 16), no statistically significant increases in chromosomal aberration frequencies were observed at any tested concentration (625, 312.5, or 156.3 μg/mL) compared with the negative control (*p* > 0.05).

Collectively, these results demonstrate that, within the tested dose ranges, exposure durations, and metabolic activation conditions, *A. cristatus* CCNH008 spore powder did not induce a significant increase in chromosomal aberrations in Chinese hamster lung (CHL) cells.

## 4. Discussion

The present study provides a strain-level safety evaluation of *A. cristatus* CCNH008. In the acute oral toxicity study, no mortality, no bodyweight loss, and no treatment-related pathological changes were observed in mice or rats, and the mild signs seen in rats shortly after dosing were temporary and resolved on their own. In the 90-day repeated oral toxicity study, no test item–related changes were found in clinical signs, bodyweight, food consumption, urinalysis, organ weights, or histopathology during either the treatment period or the 28-day recovery period. These findings support the view that CCNH008 has a low acute toxicity profile and does not cause clear subchronic systemic toxicity under the tested conditions.

The few statistically significant changes observed in serum chemistry and hematology were small, isolated, and not persistent. Minor differences were detected at a few time points in albumin, triglycerides, sodium, neutrophil percentage, monocyte percentage, prothrombin time, and lymphocyte percentage; however, these changes did not show a clear dose–response relationship and were not accompanied by changes in organ weights or histopathological lesions. According to EFSA guidance, statistical significance alone does not indicate biological relevance; the magnitude, pattern, and consistency of the response must also be considered [29]. In the present study, the scattered nature of these changes and the absence of related tissue injury suggest normal biological variation rather than treatment-related toxicity. This interpretation is consistent with EFSA safety evaluations of Aspergillus-derived food enzymes and with genomic evidence that *A. cristatus* lacks known carcinogen-producing gene clusters.

Among the altered parameters, the slight decreases in albumin and triglycerides did not indicate hepatic injury, as liver toxicity is usually supported by a broader pattern of change, such as consistent enzyme elevations together with histological lesions. No such linked response was observed here. Likewise, the transient changes in neutrophil and lymphocyte percentages were not associated with inflammatory lesions or other tissue abnormalities, making an immune-related toxic effect unlikely. From a toxicological perspective, these findings are more consistent with normal physiological fluctuation than with organ toxicity, especially because the changes were limited in magnitude and did not persist across sampling points. Similar patterns have been reported in safety studies of other food-associated microorganisms and food enzymes, where small and isolated statistical differences were considered non-adverse in the absence of supporting pathological findings. The negative genotoxicity results further support the safety profile of CCNH008. The strain did not induce mutations in the bacterial reverse mutation assay, did not increase micronucleated erythrocytes in mice, and did not cause chromosomal aberrations in CHL cells. These results are consistent with previous safety evaluations of food-associated *Aspergillus* strains. For example, EFSA assessments of food enzymes produced by non-genetically modified *Aspergillus niger* and *Aspergillus oryzae* strains reported no genotoxic concern and no adverse effects in 90-day rat studies at the highest doses tested, with NOAELs at the top dose levels [30,31,32,33]. In addition, a comparative genomic study of *A. cristatus* found no gene clusters associated with carcinogen production and concluded that the fungus is considered safe under both low- and high-osmolarity conditions [34]. Taken together, CCNH008 falls within the general safety range reported for food-associated *Aspergillus* strains used in industrial applications.

Compared with toxigenic members of the genus, such as *A. flavus* and *A. parasiticus*, which are important producers of aflatoxins in contaminated foods, *A. cristatus* is generally regarded as a beneficial fungus associated with traditional dark tea fermentation. Nevertheless, because safety characteristics may vary among strains within the same species, strain-level toxicological assessment remains necessary before broader food application. The present findings place CCNH008 within the expected safety range of food-associated fermentation microorganisms and provide toxicological evidence supporting its potential use in food-related products.

Overall, the findings from this study show that *A. cristatus* CCNH008 carries no risk of acute oral toxicity, subchronic oral toxicity, or genotoxicity under the tested conditions. As interest in functional fermented foods continues to grow, it is critical to carry out systematic safety evaluations at the strain level. The results of this study therefore provide supporting evidence for the potential safe use of CCNH008 in food products for human consumption.

While our current findings confirm a favorable safety profile for this strain, further studies are needed to complete a full safety assessment. This includes testing for reproductive and developmental toxicity, as well as studies using non-rodent species. These follow-up studies will provide additional evidence to support the safety of CCNH008 for wider use.

## Figures and Tables

**Table 1:** Positive mutagens for each tester strain.

S_9_ Condition	Strain (s)	Positive Mutagen	Final Dose
−S_9_	TA97a	ICR-191	1 µg/plate
	TA98	4-Nitroquinoline N-oxide (4-NQO)	0.5 µg/plate
	TA100, TA102	Methyl methanesulfonate (MMS)	1 µL/plate
	TA1535	Sodium azide	1.5 µg/plate
+S_9_	TA97a, TA98, TA100	2-Aminofluorene	10 µg/plate
	TA102	1,8-Dihydroxyanthraquinone	50 µg/plate
	TA1535	2-Aminoanthracene	5 µg/plate

**Table 2:** Ingredients of positive control.

S_9_	Positive Control	Solvent	Final Concentration	Dose
−S_9_	Mitomycin C	DMSO	0.25 µg/mL (4 h)	25 µL
			0.1 µg/mL (4 h)	25 µL
+S_9_	Cyclophosphamide	DMSO	20 µg/mL	25 µL

**Table 3:** Body weight changes and mortality in mice during the acute oral toxicity assay, administering a suspension of spore powder.

Sex	Dosage (mg/kg)	Number	Bodyweight Mean ± SD (g)			Relative Weight Gain (%)	Number of Deaths	Mortality Rate (%)
			0 d	7 d	14 d	(Day 14 vs. Day 0)		
Female	10,000	10	20.4 ± 1.1	27.3 ± 1.1 **	31.6 ± 1.5 **	54.9 ± 8.2	0	0
Male	10,000	10	20.5 ± 1.1	32.9 ± 1.4 **	39.0 ± 1.6 **	90.2 ± 9.5	0	0

Note: ** (*p* < 0.01) indicates a significant difference compared with Day 0; statistical analysis was performed using paired *t*-test.

**Table 4:** Body weight changes and mortality in rats during the acute oral toxicity assay, administering a suspension of spore powder.

Sex	Dosage (mg/kg)	Number	Bodyweight Mean ± SD (g)			Relative Weight Gain (%)	Number of Deaths	Mortality Rate (%)
			0 d	7 d	14 d	(Day 14 vs. Day 0)		
Female	10,000	10	185.0 ± 4.0	226.7 ± 11.5 **	246.6 ± 15.3 **	33.3 ± 6.8	0	0
Male	10,000	10	199.4 ± 7.3	279.2 ± 11.2 **	344.3 ± 16.8 **	72.7 ± 9.2	0	0

Note: ** (*p* < 0.01) indicates a significant difference compared with Day 0; statistical analysis was performed using paired *t*-test.

**Table 5:** Observation table of toxic signs in rats after exposure during the acute oral toxicity assay, administering a suspension of spore powder.

Dosage (mg/kg)	Sex	ID	0 d (0.5 h)	0 d (1 h)	0 d (2 h)	0 d (4 h)	1 w (1 d)	1 w (2 d)	1 w (3 d)	1 w (4 d)	1 w (5 d)	1 w (6 d)	1 w (7 d)	2 w (8 d)	2 w (9 d)	2 w (10 d)	2 w (11 d)	2 w (12 d)	2 w (13 d)	2 w (14 d)	Gross Anatomical Finding
10,000	Female	1	3/4	3/4	3/4	3	1	1	1	1	1	1	1	1	1	1	1	1	1	1	Normal
		2	3	3	3	3	1	1	1	1	1	1	1	1	1	1	1	1	1	1	Normal
		3	3	3	3	3	1	1	1	1	1	1	1	1	1	1	1	1	1	1	Normal
		4	3/5	3/5	3	3	1	1	1	1	1	1	1	1	1	1	1	1	1	1	Normal
		5	3	3	3	3	1	1	1	1	1	1	1	1	1	1	1		1	1	Normal
		6	3	3	3	3	1	1	1	1	1	1	1	1	1	1	1	1	1	1	Normal
		7	3/4	3/4	3/4	3	1	1	1	1	1	1	1	1	1	1	1	1	1	1	Normal
		8	3	3	3	3	1	1	1	1	1	1	1	1	1	1	1	1	1	1	Normal
		9	3	3	3	3	1	1	1	1	1	1	1	1	1	1	1	1	1	1	Normal
		10	3	3	3	3	1	1	1	1	1	1	1	1	1	1	1	1	1	1	Normal
10,000	Male	1	3/4/5	3/4/5	3/4	3	1	1	1	1	1	1	1	1	1	1	1	1	1	1	Normal
		2	3	3	3	3	1	1	1	1	1	1	1	1	1	1	1	1	1	1	Normal
		3	3	3	3	3	1	1	1	1	1	1	1	1	1	1	1	1	1	1	Normal
		4	3/5	3/5	3	3	1	1	1	1	1	1	1	1	1	1	1	1	1	1	Normal
		5	3/4/5	3/4/5	3/4	3	1	1	1	1	1	1	1	1	1	1	1	1	1	1	Normal
		6	3/4/5	3/4/5	3/4	3	1	1	1	1	1	1	1	1	1	1	1	1	1	1	Normal
		7	3	3	3	3	1	1	1	1	1	1	1	1	1	1	1	1	1	1	Normal
		8	3/4/5	3/4/5	3/4	3	1	1	1	1	1	1	1	1	1	1	1	1	1	1	Normal
		9	3	3	3	3	1	1	1	1	1	1	1	1	1	1	1	1	1	1	Normal
		10	3	3	3	3	1	1	1	1	1	1	1	1	1	1	1	1	1	1	Normal

Note: 1—no clinical signs were observed; 2—mortality; 3—reduced activity; 4—ptosis; 5—salivation.

**Table 6:** Absolute organ weights of rats at the end of the study of the 90-day oral toxicity study, administering a suspension of spore powder ( $\bar{x}$ ± SD, *n* = 10).

Sex	Male				Female			
Group	Control	Low Dose	Mid Dose	High Dose	Control	Low Dose	Mid Dose	High Dose
Fasted BW (g)	514.2 ± 65.5	469.1 ± 31.8	458.4 ± 38.5	457.2 ± 41.0	259.8 ± 21.6	272.6 ± 28.7	253.9 ± 23.0	255.2 ± 22.8
Brain (g)	2.10 ± 0.10	2.05 ± 0.11	2.06 ± 0.12	2.02 ± 0.12	1.87 ± 0.06	1.92 ± 0.08	1.87 ± 0.14	1.89 ± 0.08
Heart (g)	1.85 ± 0.32	1.67 ± 0.14	1.75 ± 0.32	1.75 ± 0.31	1.05 ± 0.15	1.09 ± 0.11	1.13 ± 0.16	1.00 ± 0.13
Thymus (mg)	571 ± 77	525 ± 94	496 ± 81	459 ± 102	342 ± 78	399 ± 93	336 ± 90	335 ± 95
Adrenal (mg)	80 ± 16	73 ± 9	78 ± 22	75 ± 12	72 ± 13	82 ± 15	80 ± 13	86 ± 7
Liver (g)	12.86 ± 2.88	12.01 ± 1.29	11.95 ± 1.35	12.25 ± 1.25	7.39 ± 1.01	7.79 ± 0.88	7.34 ± 1.58	7.95 ± 1.02
Kidney (g)	3.04 ± 0.39	2.93 ± 0.22	2.81 ± 0.40	2.88 ± 0.30	1.66 ± 0.24	1.75 ± 0.22	1.65 ± 0.20	1.68 ± 0.22
Spleen (g)	0.78 ± 0.11	0.75 ± 0.12	0.73 ± 0.09	0.73 ± 0.09	0.41 ± 0.05	0.47 ± 0.03	0.42 ± 0.06	0.43 ± 0.07
Testes/Uterus (g)	3.55 ± 0.34	3.46 ± 0.22	3.43 ± 0.30	3.40 ± 0.22	0.66 ± 0.16	0.89 ± 0.30	0.80 ± 0.20	0.81 ± 0.22
Epididymis/Ovary (mg)	1427 ± 240	1424 ± 219	1331 ± 166	1337 ± 119	153 ± 43	177 ± 54	157 ± 37	149 ± 38

Note: No significant difference was observed between any dose groups and control groups; statistical analysis was performed using an independent-samples *t*-test.

**Table 7:** Absolute organ weights of rats at the end of recovery observation of the 90-day oral toxicity study, administering a suspension of spore powder ( $\bar{x}$ ± SD, *n* = 5).

Sex	Male		Female	
Group	Control	High Dose	Control	High Dose
Fasted BW (g)	371.0 ± 16.8	347.0 ± 46.9	216.6 ± 11.8	211.0 ± 13.8
Brain (g)	2.09 ± 0.07	2.16 ± 0.05	1.89 ± 0.07	1.84 ± 0.18
Heart (g)	1.78 ± 0.10	1.77 ± 0.17	1.12 ± 0.14	1.11 ± 0.13
Thymus (mg)	425 ± 162	420 ± 115	373 ± 97	325 ± 92
Adrenal (mg)	67 ± 16	65 ± 14	77 ± 6	87 ± 27
Liver (g)	12.53 ± 0.52	12.83 ± 1.74	7.69 ± 1.18	7.99 ± 0.99
Kidney (g)	3.01 ± 0.32	3.08 ± 0.23	1.92 ± 0.37	1.92 ± 0.14
Spleen (g)	0.80 ± 0.04	0.83 ± 0.13	0.49 ± 0.07	0.47 ± 0.08
Testes/Uterus (g)	3.60 ± 0.19	3.31 ± 0.73	0.87 ± 0.22	1.01 ± 0.40
Epididymis/Ovary (mg)	1638 ± 152	1438 ± 213	177 ± 48	159 ± 39

Note: No significant difference was observed between the high dose and control groups; statistical analysis was performed using an independent-samples *t*-test.

**Table 8:** Summary of histopathological examination results in the 90-day oral toxicity study.

Organ and Microscopic Finding	Number of Affected Male Rats		Number of Affected Female Rats	
	High Dose *n* = 10	Control *n* = 10	High Dose *n* = 10	Control *n* = 10
Pituitary—adenohypophysis, cyst in distal part	1	1	0	0
Adrenal—diffuse cortical vacuolation increased	0	1	0	0
Pancreas—exocrine part, acinar atrophy with mononuclear cell infiltration	0	1	0	0
Stomach—glandular stomach, submucosal edema	0	0	1	0
Stomach—glandular stomach, glandular cyst	0	0	0	1
Kidney—cortical part, renal tubular cyst	1	0	0	0
Ovary—number of corpora lutea reduced/absent	/	/	3	2
Ovary—follicular cyst	/	/	1	1
Prostate—interstitium, mononuclear inflammatory cell infiltration	0	1	/	/
Organs/tissues with no remarkable lesions	Brain, thyroid, lungs, thymus, spleen, lymph node, heart, liver, duodenum, jejunum, ileum, colon, rectum, bladder, testes, epididymis, uterus			

**Table 9:** First bacterial reverse mutation assay (Ames test).

Dosage (µg)	Revertant Colonies for Strain									
	TA97a		TA98		TA100		TA102		TA1535	
	−S_9_	+S_9_	−S_9_	+S_9_	−S_9_	+S_9_	−S_9_	+S_9_	−S_9_	+S_9_
25	111 ± 4	123 ± 9	33 ± 3	33 ± 5	116 ± 11	139 ± 8	281 ± 18	285 ± 13	12 ± 4	11 ± 4
79	113 ± 4	122 ± 8	32 ± 2	30 ± 3	123 ± 11	121 ± 10	294 ± 19	297 ± 10	8 ± 4	13 ± 1
250	112 ± 5	120 ± 10	31 ± 2	34 ± 3	116 ± 10	129 ± 11	288 ± 22	304 ± 13	10 ± 5	11 ± 7
790	115 ± 10	112 ± 7	35 ± 5	31 ± 4	121 ± 5	120 ± 13	284 ± 16	283 ± 11	13 ± 2	8 ± 4
2500	117 ± 14	126 ± 11	32 ± 5	32 ± 2	125 ± 7	124 ± 9	286 ± 18	302 ± 13	9 ± 4	11 ± 3
Untreated control	113 ± 12	130 ± 11	30 ± 2	33 ± 6	123 ± 9	119 ± 5	282 ± 12	306 ± 12	10 ± 3	7 ± 3
Solvent control	111 ± 5	125 ± 7	30 ± 4	29 ± 4	110 ± 8	122 ± 10	296 ± 9	304 ± 14	7 ± 1	8 ± 5
Positive control	1969 ± 157	2213 ± 133	278 ± 24	3376 ± 212	2313 ± 178	2379 ± 133	3339 ± 204	1211 ± 102	891 ± 93	273 ± 21

Note: TA97a, TA98, TA100, TA102, and TA1535 are standard strains of *Salmonella typhimurium* used in the Ames test for detecting frameshift and base-pair substitution mutations.

**Table 10:** Second bacterial reverse mutation assay (Ames test).

Dosage (µg)	Revertant Colonies for Strain									
	TA97a		TA98		TA100		TA102		TA1535	
	−S_9_	+S_9_	−S_9_	+S_9_	−S_9_	+S_9_	−S_9_	+S_9_	−S_9_	+S_9_
4	109 ± 8	123 ± 11	30 ± 3	36 ± 4	119 ± 7	124 ± 11	288 ± 22	298 ± 7	9 ± 2	11 ± 1
20	118 ± 4	128 ± 12	33 ± 4	37 ± 4	122 ± 12	124 ± 6	292 ± 20	300 ± 19	7 ± 5	10 ± 3
100	112 ± 7	119 ± 8	32 ± 5	32 ± 4	120 ± 5	116 ± 10	290 ± 13	299 ± 8	12 ± 4	11 ± 6
500	118 ± 12	125 ± 8	31 ± 3	33 ± 2	116 ± 6	119 ± 11	298 ± 19	294 ± 11	11 ± 2	12 ± 4
2500	117 ± 11	119 ± 5	35 ± 5	34 ± 7	125 ± 9	122 ± 7	289 ± 18	302 ± 14	10 ± 2	13 ± 3
Untreated control	112 ± 5	128 ± 2	31 ± 2	33 ± 7	124 ± 5	125 ± 13	287 ± 12	293 ± 15	10 ± 6	9 ± 4
Solvent control	115 ± 12	121 ± 10	34 ± 3	30 ± 3	113 ± 11	128 ± 12	283 ± 20	300 ± 4	10 ± 3	8 ± 1
Positive control	1875 ± 149	2231 ± 130	284 ± 7	3384 ± 212	2337 ± 137	2480 ± 114	3412 ± 200	1160 ± 112	1054 ± 110	278 ± 13

**Table 11:** Mammalian erythrocyte micronucleus test.

Group	Dosage	Sex	Number	PCEs Scored	Micronucleated PCEs	PCE/TE Ratio (%)	MN Cell Frequency (‰)
Test group	1.67 g/kg	Female	5	10,000	3.00 ± 1.58	54.80 ± 1.44	1.50 ± 0.79
		Male	5	10,000	3.00 ± 1.58	54.20 ± 1.52	1.50 ± 0.79
	0.84 g/kg	Female	5	10,000	3.20 ± 1.30	54.60 ± 1.64	1.60 ± 0.65
		Male	5	10,000	3.20 ± 1.30	54.30 ± 2.36	1.60 ± 0.65
	0.42 g/kg	Female	5	10,000	3.20 ± 1.30	54.30 ± 1.92	1.60 ± 0.65
		Male	5	10,000	3.00 ± 1.58	54.00 ± 1.70	1.50 ± 0.79
Solvent control	1% Tween 80	Female	5	10,000	3.40 ± 1.14	54.60 ± 1.39	1.70 ± 0.57
		Male	5	10,000	3.00 ± 1.58	54.20 ± 1.79	1.50 ± 0.79
Positive control	40 mg/kg Cyclophosphamide	Female	5	10,000	43.40 ± 3.05	48.60 ± 2.77	21.70 ± 1.52 *
		Male	5	10,000	40.00 ± 2.35	48.80 ± 2.41	20.00 ± 1.17 *

Note: * (*p* < 0.05) indicates a significant difference compared with the solvent control group; statistical analysis was performed using Poisson regression with the total number of cells scored as the offset variable. PCEs = polychromatic erythrocytes; TE = total erythrocytes; MN = micronucleus.

**Table 12:** Preliminary test of cytotoxicity assay.

Group	Cells Counted	Scored Metaphases		Mitotic Index (%)	
		−S_9_	+S_9_	−S_9_	+S_9_
Negative control	500	81	84	/	/
5000	500	0	0	0	0
2500	500	0	13	0	15.5
1250	500	38	41	46.9	48.8
625	500	55	63	67.9	75.0
312.5	500	68	74	84.0	88.1

**Table 13:** Result of the in vitro mammalian chromosomal aberration assay (−S_9_ 4 h).

	Chromosomal Aberration Types												
Group (µg/mL)	Gap	Break	Fragment	Ring	Triradial	Quadriradial	Minute	Polyploidy	Pulverization	Cells Scored	Aberrant Cells	Aberration Frequency (%)	*p* Value
Negative group	0	0	0	0	0	0	0	0	0	100	0	0	/
312.5	0	2	0	0	0	0	0	0	0	100	2	2	>0.05
625	0	1	0	0	2	0	0	0	0	100	2	2	>0.05
1250	0	2	0	0	0	0	0	0	0	100	2	2	>0.05
0.25	0	4	4	7	17	5	0	0	0	100	30	30	<0.01 **

Note: ** (*p* < 0.01) indicates significant difference compared with the negative control group; statistical analysis was performed using a chi-square (χ^2^) test.

**Table 14:** Result of the in vitro mammalian chromosomal aberration assay (+S_9_ 4 h).

	Chromosomal Aberration Types												
Group (µg/mL)	Gap	Break	Fragment	Ring	Triradial	Quadriradial	Minute	Polyploidy	Pulverization	Cells Scored	Aberrant Cells	Aberration Frequency (%)	*p* Value
Negative group	0	0	0	0	0	0	0	0	0	100	0	0	/
312.5	0	0	0	1	0	0	0	0	0	100	1	1	>0.05
625	0	0	0	0	0	0	0	0	0	100	0	0	>0.05
1250	0	1	0	0	0	0	0	0	0	100	1	1	>0.05
20	0	2	3	7	17	15	0	0	0	100	28	28	<0.01 **

Note: ** (*p* < 0.01) indicates significant difference compared with the negative control group; statistical analysis was performed using a chi-square (χ^2^) test.

**Table 15:** Results of the preliminary cytotoxicity assay (−S_9_, 24 h).

Group (µg/mL)	Cells Counted	Scored Metaphases	Mitotic Index (%)
Negative	500	82	/
1250	500	24	29.3
625	500	39	47.6
312.5	500	55	67.1
156.3	500	76	92.7

**Table 16:** Results of the cytotoxicity assay (−S_9_, 24 h).

	Chromosomal Aberration Types												
Group (µg/mL)	Gap	Break	Fragment	Ring	Triradial	Quadriradial	Minute	Polyploidy	Pulverization	Cells Scored	Aberrant Cells	Aberration Frequency (%)	*p* Value
Negative group	0	0	0	0	0	0	0	0	0	100	0	0	/
156.3	0	1	0	1	0	1	0	0	0	100	3	3	>0.05
312.5	0	1	0	0	0	0	0	0	0	100	1	1	>0.05
625	0	2	0	0	1	0	0	0	0	100	2	2	>0.05
0.1	0	5	9	2	17	10	0	0	0	100	28	28	<0.01 **

Note: ** (*p* < 0.01) indicates significant difference compared with the negative control group; statistical analysis was performed using a chi-square (χ^2^) test.

## Data Availability

The original contributions presented in this study are included in the article/Supplementary Materials. Further inquiries can be directed to the corresponding author.

## Supplementary Materials

The following supporting information can be downloaded at: https://www.mdpi.com/article/10.3390/foods15122066/s1, Table S1: Body weight, body weight gain, food consumption, and food utilization efficiency in male rats during the 90-day oral toxicity study administering a suspension of spore powder; Table S2: Body weight, body weight gain, food consumption, and food utilization efficiency in female rats during the 90-day oral Toxicity study administering a suspension of spore powder; Table S3: Body weight changes in recovery observation group rats during the 90-day oral toxicity study administering a suspension of spore powder (g); Table S4: Mid-term biochemical test results of the 90-day oral toxicity study administering a suspension of spore powder; Table S5: End-of-study blood biochemical test results of the 90-day oral toxicity study administering a suspension of spore powder; Table S6: End of recovery observation blood biochemical test results of the 90-day oral toxicity study administering a suspension of spore powder; Table S7: Mid-term hematology test results of the 90-day oral toxicity study administering a suspension of spore powder; Table S8: End-of-study hematology test results of the 90-day oral toxicity study administering a suspension of spore powder; Table S9: End of recovery observation hematology test results of the 90-day oral toxicity study administering a suspension of spore powder; Table S10: Mid-term rat urinalysis results of the 90-day oral toxicity study administering a suspension of spore powder; Table S11: End-of-study rat urinalysis results of the 90-day oral toxicity study administering a suspension of spore powder; Table S12: End of recovery observation rat urinalysis results of the 90-day oral toxicity study administering a suspension of spore powder.
