# Correction: Jiao et al. Safety Assessment of *Aspergillus cristatus* CCNH008 for Potential Use in Food and Health Applications. *Foods* 2026, *15*, 2066

**DOI:** 10.3390/foods15172964

**Published:** 2026-08-24

**Authors:** Zishan Jiao, Jiahao Huang, Juan Yang, Xinyi Chen, Xiaowei Zheng

**Affiliations:** Nutrition & Health Research Institute, COFCO Corporation, Beijing 102209, China; jiaozishan@cofco.com (Z.J.); huangjiahao@cofco.com (J.H.);

## Text Correction

There were errors in the original publication [1]. The manuscript describes an incorrect version of the SPSS software and lacks clarification that the data analysis for the Mammalian erythrocyte micronucleus assay and the in vitro mammalian cell chromosomal aberration assay were performed by an external institution.

In the Methods section, Paragraph 17, the following correction has been made: “Statistical analyses were performed using SPSS Statistics, version 29.0 (IBM Corp., Armonk, NY, USA) by the Shanghai Municipal Center for Disease Control and Prevention (Shanghai Academy of Preventive Medicine). The frequencies of micronucleated PCEs in each treatment group were analyzed based on a Poisson distribution and compared with the vehicle control group.”

In the Methods section, Paragraph 20, the following correction has been made: “As no positive results were observed in the initial assays, an additional extended exposure assay without metabolic activation was performed. Cells were treated for 24 h at concentrations of 625, 312.5, and 156.3 μg/mL, as determined from the pretest. The experimental procedures and evaluations were conducted as described above. Statistical analyses were performed using SPSS Statistics, version 29.0 (IBM Corp., Armonk, NY, USA) by the Shanghai Municipal Center for Disease Control and Prevention (Shanghai Academy of Preventive Medicine). The incidence of cells with structural chromosomal aberrations (excluding gaps) was compared between treatment groups and the negative control using the χ^2^ test or Fisher’s exact test, where appropriate. A Cochran–Armitage trend test was applied to assess the presence of a dose–response relationship. All statistical tests were two-sided, and *p* < 0.05 was considered statistically significant.”

The authors state that the scientific conclusions are unaffected. This correction was approved by the Academic Editor. The original publication has also been updated.
