# Supplementary material for: Safety Assessment of Aspergillus cristatus CCNH008 for Potential Use in Food and Health Applications
Source: Foods. 2026 Jun 8;15(12):2066. doi: 10.3390/foods15122066 (PMC13298308; doi:10.3390/foods15122066)
Supplement: Supplementary file 1 [file foods-15-02066-s001.zip › foods-4290308-supplementary.pdf]

Supplementary Tables

Supplementary Table S1: Body Weight, Body Weight Gain, Food Consumption, and Food Utilization Efficiency in Male Rats during the 90-Day Oral Toxicity study administering a suspension of spore powder

Time (week)	Group	Weight (g)	Food consumption (g)	Body weight gain (g)	Food utilization efficiency (g)
Initial body weight	Vehicle	66.6±7.0	/	/	/
	Low-dose	66.5±6.0			
	Mid-dose	66.9±5.4			
	High-dose	67.1±5.0			
1	Vehicle	118.6 ± 14.2	109.3 ± 15.2	52.0 ± 8.2	47.6 ± 2.8
	Low-dose	112.6 ± 10.5	103.5 ± 11.1	46.1 ± 10.4	45.4 ± 10.7
	Mid-dose	112.2 ± 7.3	99.8 ± 6.8	45.3 ± 5.3	45.4 ± 3.9
	High-dose	110.4 ± 8.6	97.0 ± 10.2	43.3 ± 6.7	44.5 ± 3.2
2	Vehicle	179.1 ± 17.2	156.4 ± 15.1	60.5 ± 8.4	38.7 ± 3.5
	Low-dose	177.2 ± 9.7	146.8 ± 10.1	64.6 ± 14.4	43.8 ± 7.5
	Mid-dose	169.9 ± 14.2	137.9 ± 13.1**	57.7 ± 8.8	41.7 ± 3.4
	High-dose	164.2 ± 12.4	137.8 ± 13.3**	53.8 ± 11.2	38.7 ± 5.8
3	Vehicle	242.1 ± 25.7	175.8 ± 17.1	63.0 ± 9.8	35.8 ± 2.3
	Low-dose	235.1 ± 12.0	168.9 ± 11.5	57.9 ± 6.3	34.3 ± 2.5
	Mid-dose	227.9 ± 16.0	165.8 ± 13.8	58.0 ± 5.2	35.1 ± 2.4
	High-dose	220.7 ± 13.9	156.4 ± 11.8**	56.5 ± 5.3	36.3 ± 2.6
4	Vehicle	302.3 ± 31.0	188.8 ± 19.8	60.2 ± 9.1	32.1 ± 3.7
	Low-dose	290.0 ± 14.7	182.3 ± 9.5	54.9 ± 7.0	30.1 ± 3.6
	Mid-dose	278.6 ± 16.7*	178.9 ± 14.4	50.7 ± 4.5	28.3 ± 2.9
	High-dose	270.4 ± 16.1**	170.8 ± 10.5	49.7 ± 6.4*	29.0 ± 3.1
5	Vehicle	356.9 ± 41.7	206.0 ± 24.2	54.6 ± 12.4	26.2 ± 3.4
	Low-dose	336.2 ± 15.2	189.7 ± 9.2	46.2 ± 5.4	24.2 ± 2.7
	Mid-dose	325.3 ± 20.8	191.4 ± 16.8	46.7 ± 5.5	24.4 ± 1.8
	High-dose	318.7 ± 19.6	185.6 ± 12.8	48.3 ± 6.6	25.9 ± 2.7

6	Vehicle	393.0 ± 46.2	198.2 ± 21.6	36.1 ± 8.3	18.3 ± 3.0
	Low-dose	363.2 ± 16.6	178.3 ± 12.5*	27.0 ± 8.0	15.1 ± 3.8
	Mid-dose	356.8 ± 23.0	182.5 ± 16.5	31.5 ± 5.4	17.3 ± 2.8
	High-dose	348.7 ± 20.3	180.6 ± 15.4	30.0 ± 9.2	16.4 ± 4.4
7	Vehicle	429.7 ± 52.3	204.3 ± 26.0	36.7 ± 7.6	17.7 ± 1.9
	Low-dose	390.6 ± 20.4	184.1 ± 14.4	27.4 ± 5.8**	14.7 ± 2.2*
	Mid-dose	380.7 ± 24.9	179.1 ± 14.7	23.9 ± 4.2**	13.3 ± 2.1**
	High-dose	376.0 ± 24.5	180.2 ± 15.3	27.3 ± 6.4**	14.9 ± 2.7*
8	Vehicle	458.8 ± 55.4	200.8 ± 24.1	29.1 ± 7.1	14.5 ± 2.8
	Low-dose	422.7 ± 20.9	183.9 ± 13.1	32.1 ± 5.4	17.5 ± 2.4
	Mid-dose	411.3 ± 29.7	185.7 ± 17.9	30.6 ± 6.2	16.4 ± 2.9
	High-dose	403.1 ± 26.6	182.4 ± 19.3	27.1 ± 7.0	14.6 ± 2.7
9	Vehicle	481.0 ± 56.6	195.3 ± 20.6	22.2 ± 4.9	11.4 ± 2.3
	Low-dose	441.3 ± 27.4	181.4 ± 18.2	18.6 ± 7.9	10.0 ± 3.8
	Mid-dose	429.0 ± 33.0	180.2 ± 20.3	17.7 ± 6.8	9.7 ± 3.5
	High-dose	427.4 ± 32.3	184.6 ± 21.0	24.3 ± 8.4	13.0 ± 3.8
10	Vehicle	499.7 ± 58.9	192.7 ± 23.9	18.7 ± 9.1	9.5 ± 4.2
	Low-dose	454.6 ± 25.6	179.0 ± 13.1	13.3 ± 7.7	7.6 ± 4.6
	Mid-dose	444.4 ± 31.2	182.0 ± 22.2	15.4 ± 5.5	8.3 ± 2.7
	High-dose	440.2 ± 35.0	181.3 ± 18.6	12.8 ± 6.7	7.0 ± 3.1
11	Vehicle	516.9 ± 60.4	191.7 ± 21.2	17.2 ± 4.9	9.1 ± 2.3
	Low-dose	473.2 ± 27.1	183.5 ± 16.4	18.6 ± 5.5	10.2 ± 2.5
	Mid-dose	463.5 ± 34.6	189.6 ± 23.5	19.1 ± 6.0	10.2 ± 2.5
	High-dose	461.7 ± 35.6	189.4 ± 17.3	21.5 ± 5.6	11.4 ± 2.6
12	Vehicle	528.4 ± 64.9	197.1 ± 28.2	11.5 ± 7.7	5.5 ± 3.0
	Low-dose	483.4 ± 32.3	189.5 ± 15.9	10.2 ± 8.7	5.3 ± 3.9
	Mid-dose	475.1 ± 34.6	194.1 ± 20.9	11.6 ± 6.3	6.0 ± 3.0
	High-dose	473.4 ± 37.4	195.2 ± 20.0	11.7 ± 6.3	6.0 ± 3.1
13	Vehicle	542.8 ± 69.6	148.1 ± 20.9	14.4 ± 6.4	9.6 ± 4.0
	Low-dose	501.8 ± 36.1	144.9 ± 14.1	18.4 ± 7.1	12.7 ± 3.9
	Mid-dose	491.8 ± 40.0	144.9 ± 16.5	16.7 ± 11.2	11.1 ± 7.5
	High-dose	487.1 ± 43.2	143.2 ± 16.9	13.7 ± 8.4	9.2 ± 5.3

Note: * (P < 0.05) and ** (P < 0.05) indicate significant differences compared with the control group; statistical analysis was performed using an independent-samples *t*-test.

Supplementary Table S2: Body Weight, Body Weight Gain, Food Consumption, and Food Utilization

Efficiency in Female Rats during the 90-Day Oral Toxicity study administering a suspension of spore powder

Time (week)	Group	Weight (g)	Food consumption (g)	Body weight gain (g)	Food utilization efficiency (g)
Initial body weight	Vehicle	65.9 ± 8.8	/	/	/
	Low-dose	66.2 ± 9.1			
	Mid-dose	66.1 ± 9.0			
	High-dose	66.5 ± 8.2			
1	Vehicle	110.1 ± 9.7	100.9 ± 8.3	44.2 ± 4.6	43.8 ± 3.3
	Low-dose	110.3 ± 10.9	102.4 ± 9.2	44.1 ± 3.8	43.1 ± 4.0
	Mid-dose	107.6 ± 12.0	96.8 ± 11.1	41.5 ± 4.7	43.0 ± 2.8
	High-dose	108.9 ± 7.4	99.6 ± 7.0	42.4 ± 5.3	42.4 ± 4.2
2	Vehicle	150.8 ± 11.7	123.8 ± 13.0	40.7 ± 7.0	32.8 ± 3.7
	Low-dose	153.3 ± 11.0	123.2 ± 7.4	43.0 ± 5.7	34.9 ± 3.8
	Mid-dose	146.1 ± 14.5	121.5 ± 14.2	38.5 ± 4.7	31.6 ± 2.3
	High-dose	147.6 ± 4.9	118.4 ± 7.8	38.7 ± 8.0	32.5 ± 5.4
3	Vehicle	175.5 ± 13.7	124.2 ± 12.7	24.7 ± 5.5	19.8 ± 3.4
	Low-dose	182.9 ± 13.2	130.1 ± 13.6	29.6 ± 8.1	22.6 ± 4.8
	Mid-dose	168.8 ± 19.0	123.7 ± 12.1	22.7 ± 8.5	18.3 ± 6.1
	High-dose	176.3 ± 6.9	124.6 ± 11.0	28.7 ± 9.5	22.8 ± 5.8
4	Vehicle	200.3 ± 16.7	126.5 ± 13.9	24.8 ± 5.4	19.7 ± 3.4
	Low-dose	210.3 ± 15.0	137.1 ± 12.3	27.4 ± 6.3	19.9 ± 3.9
	Mid-dose	194.7 ± 19.7	129.4 ± 10.7	25.9 ± 5.0	20.2 ± 3.6
	High-dose	198.8 ± 10.3	127.2 ± 8.6	22.5 ± 5.2	17.7 ± 3.9
5	Vehicle	221.2 ± 20.3	134.3 ± 16.9	20.9 ± 5.6	15.4 ± 2.9
	Low-dose	233.7 ± 19.6	140.0 ± 15.8	23.4 ± 6.0	16.6 ± 2.8
	Mid-dose	216.9 ± 23.0	132.7 ± 13.4	22.2 ± 5.7	16.7 ± 3.1
	High-dose	221.2 ± 14.6	133.6 ± 8.6	22.4 ± 5.1	16.8 ± 3.4
6	Vehicle	231.4 ± 19.9	125.7 ± 15.5	10.2 ± 5.2	8.2 ± 3.6
	Low-dose	246.9 ± 22.7	136.1 ± 14.5	13.2 ± 5.5	9.6 ± 3.5
	Mid-dose	226.1 ± 23.0	127.7 ± 13.0	9.2 ± 5.9	7.1 ± 4.0

	High-dose	235.8 ± 15.2	129.0 ± 7.8	14.6 ± 4.6	11.3 ± 3.3
7	Vehicle	243.5 ± 21.1	130.1 ± 16.0	12.1 ± 5.4	9.0 ± 3.4
	Low-dose	257.4 ± 24.1	136.0 ± 13.9	10.5 ± 7.5	7.7 ± 5.6
	Mid-dose	236.8 ± 26.0	135.0 ± 13.6	10.7 ± 5.6	7.6 ± 3.7
	High-dose	242.5 ± 18.3	128.4 ± 12.7	6.7 ± 5.5	5.0 ± 3.8
8	Vehicle	256.4 ± 21.9	127.2 ± 15.0	12.9 ± 4.2	10.1 ± 3.2
	Low-dose	273.6 ± 24.7	136.3 ± 15.4	16.2 ± 3.8	12.2 ± 3.3
	Mid-dose	248.2 ± 24.9	131.7 ± 12.2	11.4 ± 5.0	8.7 ± 4.2
	High-dose	252.8 ± 19.5	130.6 ± 10.7	10.3 ± 4.8	7.9 ± 4.0
9	Vehicle	261.3 ± 24.7	120.2 ± 17.6	4.9 ± 4.9	3.8 ± 3.8
	Low-dose	277.6 ± 27.8	133.7 ± 19.9	4.0 ± 4.0	2.8 ± 2.3
	Mid-dose	256.9 ± 24.9	128.8 ± 10.9	8.7 ± 6.5	6.6 ± 4.2
	High-dose	259.6 ± 18.5	126.4 ± 11.9	6.8 ± 5.6	5.3 ± 4.2
10	Vehicle	268.5 ± 26.0	122.5 ± 17.2	7.2 ± 6.1	5.7 ± 4.7
	Low-dose	282.8 ± 29.8	129.3 ± 16.6	5.2 ± 3.9	4.0 ± 2.8
	Mid-dose	262.9 ± 25.7	126.5 ± 11.6	6.0 ± 5.8	4.7 ± 4.3
	High-dose	265.2 ± 20.5	120.1 ± 9.5	5.6 ± 5.3	4.7 ± 4.3
11	Vehicle	273.5 ± 24.0	121.7 ± 13.7	5.0 ± 3.9	4.1 ± 3.3
	Low-dose	289.3 ± 33.3	136.0 ± 18.9	6.5 ± 5.2	4.7 ± 3.1
	Mid-dose	269.5 ± 26.0	128.3 ± 20.5	6.6 ± 5.7	5.0 ± 4.1
	High-dose	272.4 ± 21.9	130.6 ± 11.6	7.2 ± 3.5	5.6 ± 2.6
12	Vehicle	277.4 ± 23.5	122.0 ± 11.9	3.9 ± 3.8	3.3 ± 3.2
	Low-dose	293.0 ± 33.4	137.4 ± 18.5	3.7 ± 2.9	2.7 ± 2.3
	Mid-dose	275.9 ± 27.1	135.9 ± 17.3	6.4 ± 5.8	4.5 ± 3.6
	High-dose	276.5 ± 20.8	128.5 ± 12.2	4.1 ± 4.1	3.3 ± 3.2
13	Vehicle	279.3 ± 23.9	91.1 ± 10.9	1.9 ± 1.9	2.1 ± 2.0
	Low-dose	298.9 ± 31.5	100.9 ± 12.4	5.9 ± 3.9	6.1 ± 4.1
	Mid-dose	281.7 ± 26.0	101.3 ± 11.4	5.8 ± 5.6	5.6 ± 5.4
	High-dose	279.3 ± 20.9	95.9 ± 8.1	2.8 ± 2.7	2.9 ± 2.9

Supplementary Table S3: Body Weight Changes in Recovery Observation Group Rats during the 90-Day Oral Toxicity study administering a suspension of spore powder (g)

**Gender**	**Male**		**Female**	
Group	Control	High Dose	Control	High Dose

Before treatment	61.8±3.7	62.6±4.5	61.0±4.7	62.0±3.3
Week 1	104.8±7.5	104.2±6.9	105.8±8.6	98.8±3.8
Week 2	159.0±12.5	164.2±8.7	158.4±17.7	140.6±6.4**
Week 3	219.2±15.4	226.8±13.1	187.8±28.7	170.2±10.3
Week 4	272.6±20.8	282.4±15.4	212.4±32.4	191.0±8.4
Week 5	321.2±27.3	331.4±15.3	231.6±35.5	209.8±9.6
Week 6	355.0±33.0	369.4±16.8	252.6±35.1	224.4±15.8
Week 7	385.2±35.4	396.6±20.1	262.2±29.8	231.2±14.1
Week 8	414.0±40.7	426.8±19.7	272.2±38.5	245.0±13.3
Week 9	432.4±41.1	448.4±20.2	280.4±40.0	249.8±15.3
Week 10	445.4±41.1	456.0±27.1	286.0±37.6	256.6±18.9
Week 11	464.6±40.9	479.0±24.7	288.4±38.1	259.4±15.4
Week 12	478.8±41.0	493.4±26.2	298.0±40.6	267.2±18.7
Week 13	494.0±42.2	511.6±24.0	299.4±36.0	270.0±18.7
Recovery Week 1	510.8±48.2	530.0±23.7	299.0±36.8	268.6±13.1
Recovery Week 2	521.0±44.5	537.2±22.7	307.6±38.7	281.0±14.0
Recovery Week 3	534.2±43.1	549.0±21.0	312.0±39.9	287.4±12.5
Recovery Week 4	540.6±42.5	563.0±21.2	317.8±41.4	291.8±11.1

Note: No significant difference was observed between the high dose and control groups; statistical analysis was performed using an independent-samples *t*-test.

Supplementary Table S4: Mid-Term Biochemical Test Results of the 90-Day Oral Toxicity study administering a suspension of spore powder ($\bar{x}$ ± SD, n=5)

**Gender**	**Male**		**Female**	
**Group**	**Control**	**High Dose**	**Control**	**High Dose**
AST (U/L)	110.5±16.4	99.7±26.3	96.9±24.2	90.1±18.1
ALT (U/L)	24.3±2.4	25.7±5.5	20.7±4.7	20.9±2.2
ALP (U/L)	194.2±46.1	238.0±32.4	120.8±17.9	116.6±38.4
TP (g/L)	58.7±0.6	57.2±2.2	65.1±4.0	60.1±3.6
Alb (g/L)	31.8±0.6	30.8±1.0	37.4±2.4	34.0±2.2*
AST (U/L)	110.5±16.4	99.7±26.3	96.9±24.2	90.1±18.1
ALT (U/L)	24.3±2.4	25.7±5.5	20.7±4.7	20.9±2.2
ALP (U/L)	194.2±46.1	238.0±32.4	120.8±17.9	116.6±38.4

TP (g/L)	58.7±0.6	57.2±2.2	65.1±4.0	60.1±3.6
Alb (g/L)	31.8±0.6	30.8±1.0	37.4±2.4	34.0±2.2*
BUN (mmol/L)	6.11±0.65	6.59±0.77	6.30±0.51	6.85±0.46
Cr (umol/L)	45.0±3.7	49.0±3.7	54.6±4.3	54.8±4.0
Glu (mmol/L)	7.19±1.28	8.79±1.59	6.89±0.43	6.84±0.20
TG (mmol/L)	0.39±0.13	0.37±0.08	0.34±0.03	0.30±0.04
TC (mmol/L)	1.58±0.46	1.65±0.35	2.14±0.42	1.81±0.32
K (mmol/L)	4.99±0.29	5.15±0.26	4.41±0.09	4.52±0.08
Na (mmol/L)	141.6±1.1	141.6±1.1	142.4±1.1	142.6±1.1
Cl (mmol/L)	103.8±1.1	104.0±1.2	105.2±2.3	106.2±1.1

Note:* ($P < 0.05$)indicates a significant difference compared with the control grou; statistical analysis was performed using an independent-samples *t*-test.

Supplementary Table S5: End-of-Study Blood Biochemical Test Results of the 90-Day Oral Toxicity study administering a suspension of spore powder ($\bar{x}$ ± SD, n=10)

**Gender**	**Male**				**Female**			
**Group**	**Control**	**Low Dose**	**Mid Dose**	**High Dose**	**Control**	**Low Dose**	**Mid Dose**	**High Dose**
AST (U/L)	157.8±24.7	141.2±19.9	176.2±18.6	150.7±24.6	156.9±90.4	148.2±80.8	159.1±125.5	143.9±27.4
ALT (U/L)	37.7±6.0	33.8±4.3	34.1±3.6	39.6±7.3	37.9±20.6	41.7±24.7	35.6±18.8	26.5±5.1
ALP (U/L)	100.1±12.3	99.3±18.6	99.1±15.0	101.2±28.9	47.8±17.9	53.5±14.3	59.8±27.0	56.1±23.3
TP (g/L)	62.6±2.1	61.8±2.3	62.3±2.1	60.8±1.6	75.7±2.9	73.1±3.9	69.7±6.6	72.2±5.8
Alb (g/L)	32.8±0.7	32.5±1.3	32.2±0.7	31.6±0.8*	42.3±1.8	40.0±2.2	38.4±3.5*	39.8±3.4
BUN (mmol/L)	6.41±0.94	6.37±0.89	7.30±0.72	6.65±0.68	7.83±1.04	7.71±1.23	7.64±1.37	6.71±0.51
Cr (umol/L)	53.0±3.6	50.4±2.8	54.0±3.0	54.4±2.4	66.5±6.7	64.1±4.7	59.9±3.6	59.6±2.9
Glu (mmol/L)	6.88±1.56	6.76±0.99	5.87±1.25	6.16±1.30	6.98±1.14	6.75±0.79	6.17±0.86	6.57±0.82
TG (mmol/L)	0.41±0.08	0.36±0.07	0.34±0.06	0.38±0.06	0.39±0.09	0.36±0.10	0.31±0.04*	0.30±0.04*
TC (mmol/L)	1.58±0.28	1.68±0.28	1.73±0.24	1.88±0.18	2.27±0.43	2.33±0.42	2.44±0.65	2.47±0.39
K (mmol/L)	5.44±0.21	5.44±0.33	5.78±0.15	5.80±0.42	4.59±0.75	4.22±0.45	4.32±0.41	4.52±0.31
Na (mmol/L)	144.3±0.8	145.4±0.8	145.4±0.8	145.1±1.0	143.9±1.7	145.9±2.0*	145.6±1.4	145.9±1.4*

Cl (mmol/L)	105.5±1.1	107.1±1.7	107.0±1.2	105.9±1.2	105.9±1.9	108.1±2.3	107.3±2.2	107.3±2.5

Note:* (P < 0.05) indicates a significant difference compared with the control group; statistical analysis was performed using an independent-samples *t*-test.

Supplementary Table S6: End of Recovery Observation Blood Biochemical Test Results of the 90-Day Oral Toxicity study administering a suspension of spore powder ($\bar{x}$ ± SD, n=5)

**Gender**	**Male**		**Female**	
**Group**	**Control**	**High Dose**	**Control**	**High Dose**
AST (U/L)	123.9±11.8	139.6±34.7	213.3±71.9	197.8±131.8
ALT (U/L)	31.7±3.4	31.8±9.8	73.1±36.1	67.5±50.9
ALP (U/L)	76.8±19.3	80.4±12.2	38.0±11.9	31.8±8.2
TP (g/L)	62.4±0.9	59.0±2.8*	72.5±2.7	73.1±4.7
Alb (g/L)	32.3±0.5	30.5±1.1*	40.6±2.1	41.1±2.3
BUN (mmol/L)	6.29±0.75	6.34±1.16	7.15±1.24	6.66±1.18
Cr (umol/L)	49.2±4.3	52.4±2.6	65.2±7.3	58.4±4.3
Glu (mmol/L)	7.49±0.54	8.48±2.62	7.08±1.33	6.98±1.08
TG (mmol/L)	0.46±0.15	0.39±0.09	0.31±0.05	0.33±0.08
TC (mmol/L)	1.64±0.40	1.69±0.21	2.21±0.45	2.70±0.49
K (mmol/L)	5.42±0.55	5.21±0.12	4.24±0.30	4.63±0.77
Na (mmol/L)	143.0±1.0	143.0±1.0	143.0±1.4	143.0±0.7
Cl (mmol/L)	105.2±0.8	105.6±1.7	106.4±1.3	106.2±0.8

Note: No significant difference was observed between the high dose and control groups; statistical analysis was performed using an independent-samples *t*-test.

Supplementary Table S7 : Mid-Term Hematology Test Results of the 90-Day Oral Toxicity study administering a suspension of spore powder ($\bar{x}$ ± SD, n=5)

**Gender**	**Male**		**Female**	
**Group**	**Control**	**High Dose**	**Control**	**High Dose**
WBC (10^9/L)	3.89±1.55	4.38±1.49	2.74±1.10	2.89±0.86
LYMPH (%)	88.1±1.9	82.4±4.8	86.4±3.6	91.9±4.0

MONO (%)	3.8±0.4	4.5±1.3	4.0±0.8	2.4±1.4
NEUT (%)	7.6±1.8	12.1±3.7*	9.0±3.2	5.4±2.4
EO (%)	0.5±0.2	0.9±0.6	0.6±0.5	0.3±0.3
BASO (%)	0.1±0.1	0.1±0.1	0.0±0.0	0.0±0.0
RBC (10^{12}/L)	7.19±0.32	7.34±0.32	6.73±0.17	6.71±0.14
HGB (g/L)	148±5	152±5	140±2	142±4
HCT (%)	40.1±1.6	41.4±1.6	37.8±0.5	38.1±0.8
PLT (10^9/L)	1175±60	1163±183	1151±92	1156±146
PT (s)	12.9±0.6	12.6±0.2	11.1±0.3	11.3±0.3
APTT (s)	25.7±0.9	25.3±1.1	23.3±1.8	23.6±1.1

Note: No significant difference was observed between the high dose and control groups; statistical analysis was performed using an independent-samples *t*-test.

Supplementary Table S8: End-of-Study Hematology Test Results of the 90-Day Oral Toxicity study administering a suspension of spore powder ($\bar{x}$ ± SD, n=10)

**Gender**	**Male**				**Female**			
**Group**	**Control**	**Low Dose**	**Mid Dose**	**High Dose**	**Control**	**Low Dose**	**Mid Dose**	**High Dose**
WBC (10^9/L)	4.51±2.04	3.71±1.73	3.21±0.88	4.69±1.69	1.85±0.88	1.61±0.74	1.67±1.43	1.87±0.55
LYMPH (%)	76.7±8.3	76.7±6.3	79.7±2.6	80.5±4.7	77.3±5.8	79.9±5.5	72.8±10.6	78.7±6.5
MONO (%)	5.4±2.4	4.9±2.0	3.5±1.0	4.2±1.5	5.1±1.4	4.3±1.7	3.2±1.9*	3.6±1.7
NEUT (%)	17.2±7.4	17.6±4.9	16.4±2.5	14.9±4.4	17.0±5.2	15.4±6.1	23.4±10.6	16.8±5.2
EO (%)	0.7±0.6	0.8±0.5	0.4±0.2	0.5±0.3	0.6±0.8	0.4±0.5	0.6±0.7	0.9±0.3
BASO (%)	0.0±0.0	0.0±0.0	0.0±0.0	0.0±0.0	0.0±0.0	0.0±0.0	0.0±0.0	0.0±0.0
RBC (10^{12}/L)	8.13±0.30	8.14±0.35	8.21±0.37	7.85±0.40	7.30±0.31	7.62±0.30	7.35±0.37	7.38±0.32
HGB (g/L)	147±4	151±6	153±6	150±7	143±5	146±6	145±5	144±6
HCT (%)	41.4±1.4	42.2±1.6	43.0±1.9	42.4±1.9	39.3±1.4	40.5±1.6	40.0±1.3	40.5±1.8

PLT (10^9/L)	829±100	818±90	846±134	861±63	849±63	840±112	705±265	878±102
PT (s)	13.9±1.1	13.2±0.7	13.0±0.5	12.8±0.8*	10.6±0.5	11.1±0.5	11.2±0.5	10.9±0.4
APTT (s)	23.6±1.5	22.7±1.0	23.6±0.9	23.4±1.3	20.4±1.1	21.0±1.2	20.1±1.1	20.8±0.9

Note: No significant difference was observed between any dose groups and control groups; statistical analysis was performed using an independent-samples *t*-test.

Supplementary Table S9: End of Recovery Observation Hematology Test Results of the 90-Day Oral Toxicity study administering a suspension of spore powder ($\bar{x}$ ± SD, n=5)

**Gender**	**Male**		**Female**	
**Group**	**Control**	**High Dose**	**Control**	**High Dose**
WBC (10^9/L)	3.67±1.77	4.15±2.07	1.08±0.35	1.49±0.73
LYMPH (%)	70.5±6.0	80.2±3.5*	77.2±6.9	78.7±4.4
MONO (%)	5.2±1.2	5.3±1.8	7.3±2.1	6.1±2.6
NEUT (%)	23.7±5.7	13.6±3.4**	14.2±4.6	14.5±2.9
EO (%)	0.6±0.2	0.9±0.3	1.3±1.0	0.7±0.8
BASO (%)	0.0±0.1	0.0±0.0	0.0±0.0	0.0±0.0
RBC (10^{12}/L)	8.95±0.29	8.61±0.27	8.05±0.56	7.97±0.19
HGB (g/L)	151±4	144±7	143±7	145±6
HCT (%)	45.7±1.0	43.7±2.1	42.3±1.8	43.0±1.9
PLT (10^9/L)	1161±111	1108±114	1013±90	1034±100
PT (s)	13.3±0.2	13.2±0.4	10.8±0.3	10.5±0.2
APTT (s)	22.3±1.0	22.8±0.6	19.3±1.5	20.5±1.4

Note: * (P < 0.05) and ** (P < 0.05) indicates a significant difference compared with the control group; statistical analysis was performed using an independent-samples t-test.

Supplementary Table S10: Mid-Term Rat Urinalysis Results of the 90-Day Oral Toxicity study administering a suspension of spore powder (n=5)

**Gender**	**Male**		**Female**	
**Group**	**Control**	**High Dose**	**Control**	**High Dose**

Appearance (Normal/Abnormal)	5/0	5/0	5/0	5/0
pH (x̄±SD)	7.6±0.5	8.0±0.0	8.0±0.0	7.8±0.4
Specific Gravity (x̄±SD)	1.029±0.005	1.026±0.013	1.029±0.009	1.022±0.007
Protein (P/N)	1/5	0/5	0/5	0/5
Occult Blood (P/N)	0/5	0/5	0/5	0/5
Glucose (P/N)	0/5	0/5	0/5	0/5

Note: No significant difference was observed between the high dose and control groups; statistical analysis was performed using an independent-samples *t*-test. Qualitative urinalysis results were recorded as P/N (positive/negative), P indicates the number of animals testing positive and N indicates the number of animals testing negative.

Supplementary Table S11: End-of-Study Rat Urinalysis Results of the 90-Day Oral Toxicity study administering a suspension of spore powder (n=10)

**Gender**	**Male**				**Female**			
**Group**	**Control**	**Low Dose**	**Mid Dose**	**High Dose**	**Control**	**Low Dose**	**Mid Dose**	**High Dose**
Appearance (Normal/Abnormal)	10/0	10/0	10/0	10/0	10/0	10/0	10/0	10/0
pH (x̄±SD)	7.9±0.3	7.9±0.3	7.9±0.3	7.7±0.5	7.1±0.5	7.3±0.5	7.2±0.8	7.0±0.4
Specific Gravity (x̄±SD)	1.019±0.005	1.021±0.006	1.023±0.009	1.018±0.012	1.023±0.012	1.026±0.011	1.032±0.012	1.025±0.013
Protein (P/N)	1/10	0/10	0/10	0/10	0/10	0/10	0/10	0/10
Occult Blood (P/N)	0/10	1/10	0/10	1/10	0/10	0/10	0/10	0/10
Glucose (P/N)	0/10	0/10	0/10	0/10	0/10	0/10	0/10	0/10

Note: No significant difference was observed between any dose groups and control groups; statistical analysis was performed using an independent-samples *t*-test.

Supplementary Table S12: End of Recovery Observation Rat Urinalysis Results of the 90-Day Oral Toxicity study administering a suspension of spore powder (n=5)

**Gender**	**Male**	**Female**

Group	Control	High Dose	Control	High Dose
Appearance (Normal/Abnormal)	5/0	5/0	5/0	5/0
pH ($\bar{x}$±SD)	7.6±0.5	8.4±0.5	6.7±0.3	6.5±0.5
Specific Gravity ($\bar{x}$±SD)	1.033±0.008	1.037±0.001	1.034±0.004	1.034±0.003
Protein (P/N)	1/5	0/5	0/5	0/5
Occult Blood (P/N)	0/5	0/5	0/5	0/5
Glucose (P/N)	0/5	0/5	0/5	0/5

Note: No significant difference was observed between the high dose and control groups; statistical analysis was performed using an independent-samples *t*-test.
